# Evaluation of a new imaging tool for use with major trauma cases in the emergency department

**DOI:** 10.1186/s12891-016-1337-8

**Published:** 2016-11-17

**Authors:** Moritz Crönlein, Konstantin Holzapfel, Marc Beirer, Lukas Postl, Karl-Georg Kanz, Dominik Pförringer, Stefan Huber-Wagner, Peter Biberthaler, Chlodwig Kirchhoff

**Affiliations:** 1 Department of Trauma Surgery, Klinikum rechts der Isar, Technical University of Munich, Germany, Ismaninger Strasse 22, 81675 Munich, Germany; 2 Department of Diagnostic and Interventional Radiology, Klinikum rechts der Isar, Technical University of Munich, Germany, Ismaninger Strasse 22, 81675 Munich, Germany; 3 Department of Oral and Maxillofacial Surgery, Kepler University Hospital, Johannes Kepler University, Krankenhausstraße 9, 4021 Linz, Austria

**Keywords:** PanCT, Whole Body CT, Trauma room management

## Abstract

**Background:**

The aim of this study was to evaluate potential benefits of a new diagnostic software prototype (Trauma Viewer, TV) automatically reformatting computed tomography (CT) data on diagnostic speed and quality, compared to CT-image data evaluation using a conventional CT console.

**Methods:**

Multiple trauma CT data sets were analysed by one expert radiology and one expert traumatology fellow independently twice, once using the TV and once using the secondary conventional CT console placed in the CT control room. Actual analysis time and precision of diagnoses assessment were evaluated. The TV and CT-console results were compared respectively, but also a comparison to the initial multiple trauma CT reports assessed by emergency radiology fellows considered as the gold standard was performed. Finally, design and function of the Trauma Viewer were evaluated in a descriptive manner.

**Results:**

CT data sets of 30 multiple trauma patients were enrolled. Mean time needed for analysis of one CT dataset was 2.43 min using the CT console and 3.58 min using the TV respectively. Thus, secondary conventional CT console analysis was on average 1.15 min shorter compared to the TV analysis.

Both readers missed a total of 11 diagnoses using the secondary conventional CT console compared to 12 missed diagnoses using the TV. However, none of these overlooked diagnoses resulted in an Abbreviated Injury Scale (AIS) > 2 corresponding to life threatening injuries.

**Conclusions:**

Even though it took the two expert fellows a little longer to analyse the CT scans on the prototype TV compared to the CT console, which can be explained by the new user interface of the TV, our preliminary results demonstrate that, after further development, the TV might serve as a new diagnostic feature in the trauma room management. Its high potential to improve time and quality of CT-based diagnoses might help in fast decision making regarding treatment of severely injured patients.

## Background

In Germany approximately 38.000 patients get severely injured by trauma per year [1, 2]. About 15% of the patients, admitted to the trauma room, die because of their traumatic injuries [1]. In the last decade the performance of computed tomography (CT) in the early trauma work-up has been identified as crucial for an early and objective detection of life threatening conditions and the consecutive initiation of treatment [3–6].

Multislice CT (MSCT) serves as an ideal imaging technique especially in the field of trauma management because of its high diagnostic precision and examination speed itself [3, 4, 7–9]. Ever since the first introduction of CT, the development of modern CT-scanners has lead to a tremendous reduction of scan time especially when it comes to whole body imaging in trauma room care [10–12]. Therefore, even in local trauma centres, a CT-scanner is mandatory according to the German Trauma Network Guidelines [1]. However, a radiologist does not need to be present on admission in these local trauma centres [1, 13]. This fact is potentially critical facing the pressure of time in trauma management with respect to the “golden hour of shock” [4, 14, 15].

Therefore, it should be mentioned that in the process of performing CT in the trauma room, the initial potential time delay is patient-related since certain time is needed to centre and prepare the patient before the CT-scan itself can be started. Later on also the time for performing multiplanar reconstructions (MPR) and sending the images to the dedicated PACS for reading might generate a certain time delay of the therapeutic regimen and finally end up in a poorer patient outcome.

In this context, Siemens Healthcare GmbH developed a new diagnostic software prototype, the so-called Trauma Viewer (TV), presenting a new diagnostic feature possibly supporting an automatic reformatting of CT images acquired during trauma room management of multiple trauma patients.

Therefore, the aim of our study was to evaluate if diagnostic speed and quality of the TV in detecting injuries in multiple trauma patients during trauma room management are comparable to the results of a conventional CT console using reformatted images performed by the CT technician.

## Methods

### Data collection

Anonymized CT data sets of patients who had suffered from multiple trauma and had been admitted to the trauma room of our University level 1 trauma centre in the years 2013 and 2014 having received a contrast enhanced MSCT scan as part of the primary survey were consecutively enrolled according to our trauma room algorithm [16].

### Setting of data analysis

**Fig. 1:**
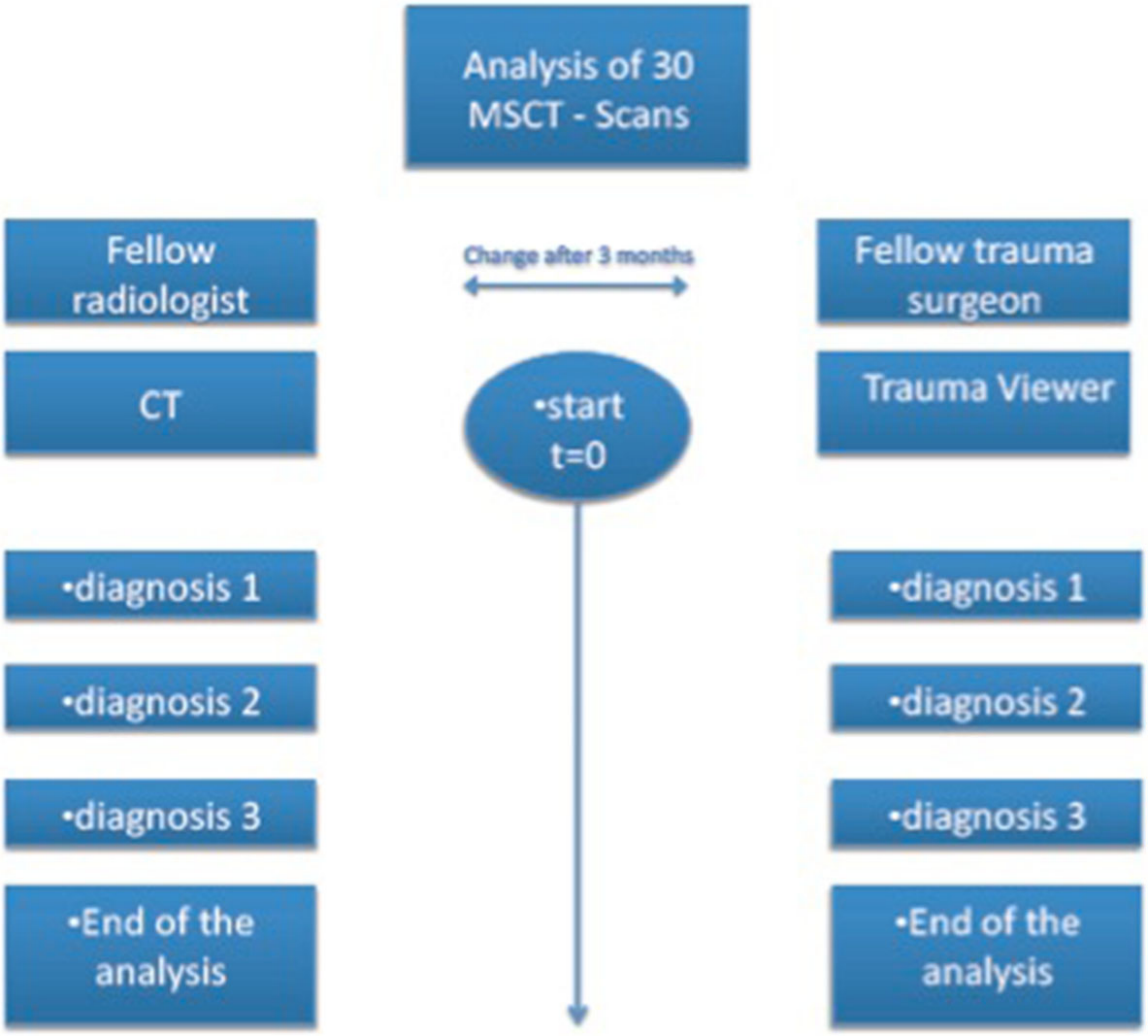
Study setting. The left image demonstrates the CT-image analysis on the secondary conventional CT console. The right image correspondingly shows the CT-image analysis using the TV respectively

In this retrospective study one board certified trauma surgery fellow with long lasting expertise in trauma management and one board certified radiology fellow also considered as long time expert in trauma imaging independently analysed the multiple trauma CT scans twice, once using the Trauma Viewer and once using the conventional secondary CT console placed in the CT control room respectively. A time interval of three months was chosen in-between both readings to prevent from certain commemorative bias using the correspondingly other modality as in the first reading (see Fig. 1).

The secondary CT console consisted of two screens whereas one was only used to upload the CT examinations from the PACS and the other screen was used to perform the image analysis itself. For the reading performed at the secondary CT console, the multiple trauma CT scans were available as initially processed for the trauma room evaluation in terms of axial images of the head, images of the spine and pelvis in all three orientations, axial images of thorax and abdomen with corresponding sagittal and coronal reformats but also available 3D-reformats as well as MIP-reformats for a better evaluation of the great vessels and parenchymal organs. Time parameters were assessed by an independent assistant using a stop clock. Time recording was started when all CT-reformats were loaded onto the conventional secondary CT console and the TV was started and ready for use respectively. Both readers were not supplied with any clinical information e.g. regarding the underlying cause for the multiple trauma CT scan in terms of trauma mechanisms. Regarding the time parameters not only the overall time needed to complete the reading of one multiple trauma CT scan using the conventional secondary CT console and the TV respectively but also the time until single diagnosis were stated, was recorded as well as the diagnoses themselves were assessed by the independent assistant.

The assessed findings/diagnoses were compared to the initially during trauma room management reported findings considered as the gold standard. The initial multiple trauma CT was read by a dedicated emergency radiology fellow with long standing experience on the secondary conventional CT console as performed in our study.

**Fig. 2:**
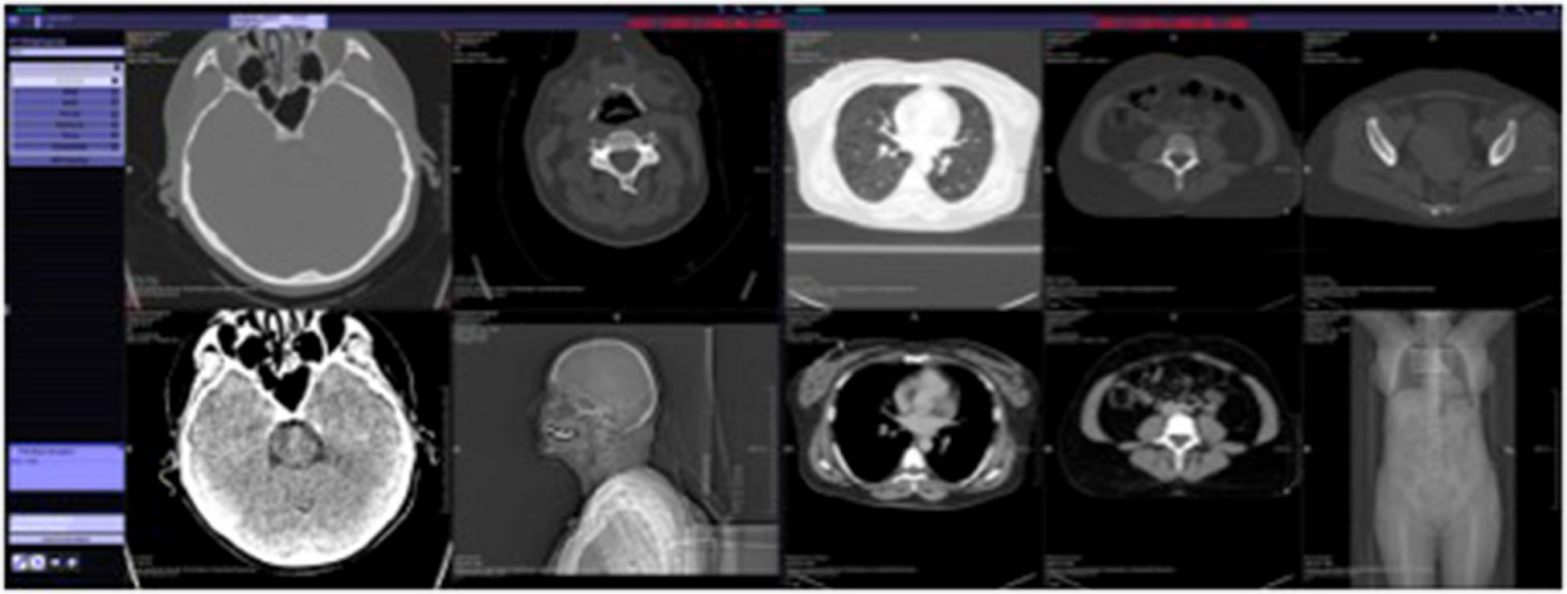
Overview of the Trauma Viewer. Display of the Trauma Viewer presenting all regions of interest from left to right: head/neck - chest - abdomen and pelvis

**Fig. 3:**
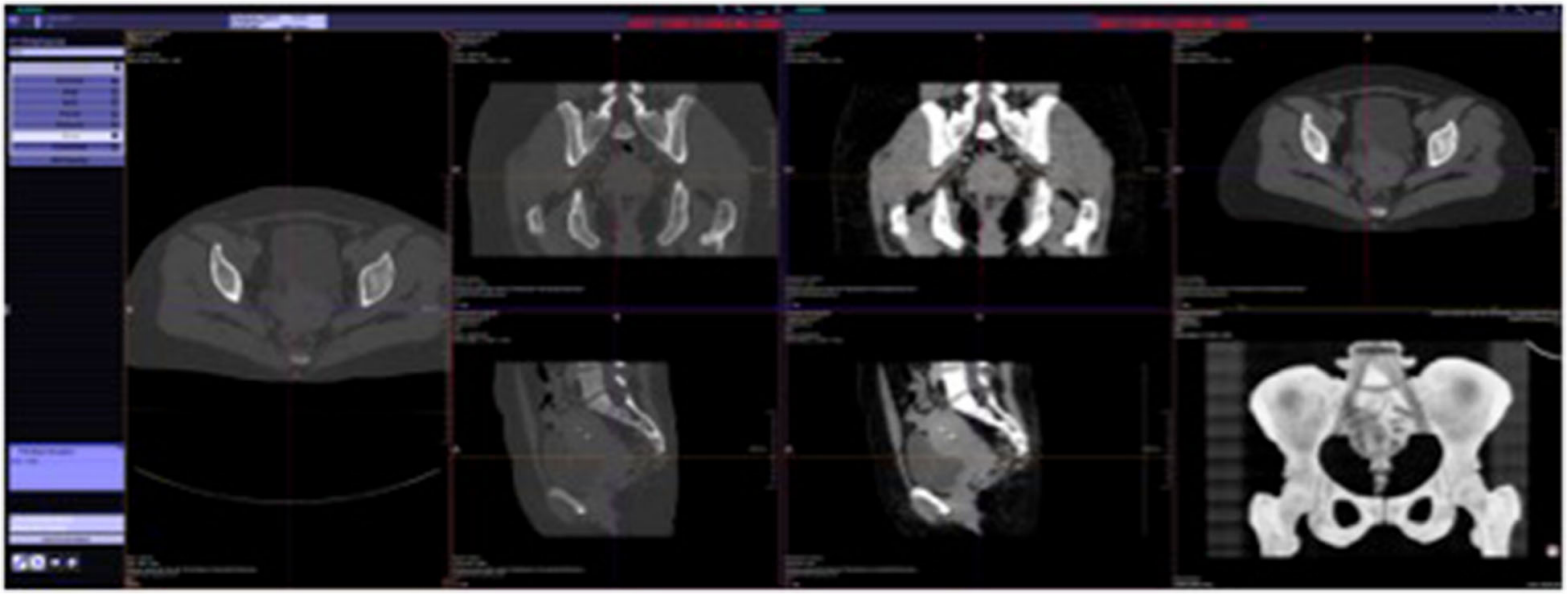
Example of Trauma Viewer results of the pelvis. The pelvic region is displayed on the TV in all offered viewing options. The axial bony image is shown on the very left side followed to the right side by coronal reformats in bone and soft tissue window in the upper row and by sagittal reformats in bone and soft tissue window in the lower row. On the right side the upper image presents the image scout and the lower image demonstrates a 3D reformat of the bony pelvis

Finally, design and functionality of the TV were evaluated in a descriptive manner. In this context Fig. 2 provides an overview of the TV itself. The left bar allows for the user to choose between the different examined body regions i.e. head/neck, chest, abdomen etc. which can be activated separately. In addition, Fig. 3 shows exemplarily whether one specific body region of interest is chosen, in this case, the pelvis which is displayed from left to right in axial, sagittal and coronal as well as in 3D orientation with the possibility to scroll through each single reconstruction separately.

### Main analysis parameters

Time until the readers defined each single diagnosis as well as the total time (in minutes) needed for analysing the entire whole body trauma CT exam were considered as primary parameters (see Fig. 1).

Diagnoses defined by both readers and in both readings respectively were analysed regarding overlooked and/or wrong diagnoses by comparing them to the gold standard were considered as secondary parameter. Consecutively the overlooked or misdiagnosed findings were categorized for their importance following the Abbreviated Injury Scale (AIS) and relevance regarding treatment and outcome.

### Statistical analysis

Data is given in mean values (arithmetic mean) and standard deviations. For the comparison of the time needed for the analysis using the TV compared to the time needed using the secondary conventional CT *console*, the paired t-test was performed using the software Sigma Stat Version 3.5 (Systat Software, Inc., San Jose, California, USA).

For statistical analysis the time results of both reading options were compared. The interrater reliability as well as the positive and negative predictive value in accordance with sensitivity and specificity were calculated using the Krippendorf’s alpha for interval data test with a level of significance of α > 0.8. To assess the interrater reliability between the trauma surgery fellow and the radiology fellow, the differences between the calculated ISS scores of the gold standard and the calculated ISS scores determined by both readers on both modalities (i.e. TV and secondary CT console) were compared.

## Results

In the evaluation period of the years 2013 and 2014 overall 30 multiple trauma CT exams performed in the trauma room of our level I University trauma centre were consecutively enrolled and analysed as described above.

Regarding the primary parameter analysis, the evaluation of the total time needed for the CT reading of all enrolled 30 whole body CT exams twice by both readers accounted for 112 min using the secondary conventional CT console with a mean of 2.433 ± 0.349 min evaluation time for one single CT and for 158 min using the TV with a mean of 3.583 ± 0.436 for one single CT-evaluation respectively. Both, the fellow trauma surgeon and the fellow radiologist, performed significantly faster in analysing the data sets on the secondary CT console compared to the TV (see Table 1). It needs to be mentioned that the CT exams enrolled showed different patterns of injury with different severity respectively.

**Table 1:** Comparison of the analysis time of the trauma surgery fellow and the radiology fellow using both reading modalities (CT and TV)

	CT	Trauma viewer	*p*-value
Fellow radiologist	2.187 ± 1.130	3.275 ± 1.339	0.001
Fellow trauma surgeon	2.680 ± 1.171	3.891 ± 1.724	0.0023
*p*-value	0.103	0.127	
Mean analysis time of both fellows	2.433 ± 0.349	3.583 ± 0.436	

This table provides the mean values and standard deviations of the analysis. *P*-values of < 0.01 were considered as statistically significant

In comparison to the initial multiple trauma CT report compiled by an experienced emergency radiology fellow considered as gold standard, there was no significant difference in the diagnostic reading accuracy between the use of the secondary conventional CT console and the TV. In total, both readers presented a rate of 11 overlooked diagnoses using the conventional secondary CT console compared to 12 overlooked diagnoses using the TV (see Table 2). However, referring to the clinical importance of these overlooked diagnoses, none of the diagnosis presented an Abbreviated Injury Scale (AIS) > 2 in terms of a greater injury severity with possible impact on the consecutive trauma treatment and outcome of the patients.

**Table 2:** Missed diagnoses with defined average abbreviated injury scale scores (AIS)

Overlooked diagnoses	CT	AIS	Trauma Viewer	AIS
intracranial haemorrhage	0		0	
maxillofacial injuries	2	2	2	2
rib fractures	3	1	4	1
lung contusion	1	2	1	2
spinal fractures	0		0	
intra-abdominal injuries	2	2	2	2
pelvic fractures	1	2	0	
other	2		3	

Overlooked diagnoses are illustrated in this table whereas 11 diagnoses were overlooked using the CT console and 12 diagnoses were overlooked using the TV respectively. The overlooked diagnoses presented with only a “minor” or “moderate” injury severity with an AIS of 1 or 2 points considered as not life-threatening conditions

The interrater reliability among the fellow trauma surgeon and the fellow radiologist using the secondary conventional CT scan accounted for α = 0.511 and turned out to be not significant. The interrater reliability between fellow trauma surgeon and fellow radiologist using the TV accounting for α = 0.855 was significantly different.

In the enrolled 30 whole body CT scans analysed, a total of 156 diagnoses were defined (see Table 3). The major findings were intracranial haemorrhage, maxillofacial fractures and spinal injuries (see Table 4).

**Table 3:** Analysis of the defined diagnoses

	CT	Trauma Viewer
patients (total)	60	60
diagnosis (total)	156	156
overlooked diagnoses	11	12
false association	2	7
false diagnosis	2	3

Overview of the defined diagnoses using conventional CT and Trauma Viewer in comparison focusing on overlooked or false diagnoses as well as on false association of diagnoses

**Table 4:** Major diagnoses

Major diagnoses	Number
intracranial haemorrhage	42
maxillofacial injuries	38
spinal fractures	32
thoracic injuries	26
intra-abdominal injuries	4
pelvic fractures	8
other	6

Most common diagnoses of the 156 found diagnoses resulting from the presented analysis are shown

Seven of the 156 assessed diagnoses were considered false positive in terms that the diagnosis was correct, but not considered as such. In three cases rib fractures were falsely associated and in the remaining four cases vertebral body fractures were falsely associated using the TV. For example, the 7th rib was described as fractured by the readers, but the gold standard described a fractured 9th rib.

Overall for the analysis using the TV, three cases with false diagnoses resulted from both readers compared to two cases with false diagnosis using the conventional secondary CT console in comparison to the gold standard (see Table 2).

Regarding the evaluation of the design and functionality Fig. 2 provides an overview of the TV itself. As the TV presents an initial prototype, minor interaction issues occurred during the evaluation. A system freezing occurred twice during the assessment, whereas it remained uncertain whether the TV software or the Windows software caused these minor problems. Besides that, there were some time delays in the windowing process at the beginning.

## Discussion

According to the German trauma-registry, 5.2% multiple trauma patients decease already during trauma room management [2]. From the socio-economical point of view trauma related death is even more important than cancer-related death or death due to cardiovascular diseases, since trauma is the leading cause of death in young adults between 15 and 35 years [1]. In this context, a lot of efforts were made in the last years to reduce trauma related death, especially regarding the optimization of the patient management in the trauma room [17]. Besides improving the training of the trauma team following the ATLS concept [18–20] and establishing well-structured guidelines for the trauma workflow [10], “time management” is still a major issue in the current literature [21–25].

To optimize the time management during trauma room care, the integration of the MSCT in the trauma room provides the opportunity to speed up the overall diagnostic process [1, 5, 9–11, 23].

Another possibility to accelerate the diagnostic workflow is reducing the time needed for establishing diagnoses in the course of the primary survey. Therefore, the presented prototype TV might serve as a future diagnostic feature in trauma room management especially for all in the initial trauma room management enrolled specialties besides radiology.

### Analysis time

However, the actual results show that it took on an average 1.15 min longer to analyse the CT scans of multiple injured patients admitted to the trauma room of our university level I trauma centre using the TV compared to using a conventional secondary CT console. There are several possible explanations for this observation: On the one hand, the readers were not used to handling the CT-exams on the TV. On the other hand, compared to the conventional secondary CT console where CT-images are available in the way they were initially processed in terms of conventional reformats of multiple trauma scans, the TV provides multiple reformats of the data with various windowing presets. The availability of additional information might result in longer reading time. Furthermore, the TV is an initial research prototype, which has not been optimized for everyday performance yet. Finally, the time recording was started when the entire CT exam including all reformats either stored in the PACS (conventional console) or performed by the TV were available for reading. The time for reformatting the axial CT raw data of the trauma CT scan which is supposed to be much faster compared to the reformats performed by the CT technician, considered as the probable real benefit of the TV, was not determined in our study. Therefore, a prospective study is necessary to assess the benefits of the TV in a real-time setup being focus of a current study of our work group.

In conclusion, additional studies recording the real time needed for reformatting the images until a diagnosis is stated have to be performed to demonstrate the advantages of the TV in time management during trauma room care of multiple injured patients.

### Quality of diagnosis

Similar to the time management during trauma room care, the quality of CT imaging is subject of a continuous improvement over the years [21, 26, 27]. Quality and speed of the MSCT scanners developed from 4 slice CT scanners in the 1990s up to 256 slice scanners nowadays resulting in higher image resolution and in the possibility to reformat 3D-images with an excellent image quality within a shorter period of time [10]. As part of our study, the diagnostic reading accuracy was evaluated as well.

Referring to a total of 156 diagnoses described in the initial polytrauma radiological reports, a total of only 12 diagnoses were overlooked by both readers using the TV, compared to 11 overlooked diagnosis using the conventional CT. Most of the overlooked diagnoses were non-displaced rib fractures and maxillofacial injuries, which were difficult to diagnose. In this context it needs to be mentioned, that none of these overlooked diagnoses had an AIS >2, so that only diagnoses with moderate injury severity were overlooked without any impact and clinical relevance on the consecutive treatment.

Using the secondary conventional CT console, the interrater reliability between the fellow traumatologist and the fellow radiologist turned out to be low. Thus the presented data provides evidence that the radiology fellow presented better results compared to the gold standard using the conventional CT console. However, the interrater reliability was comparably high using the TV, whereas here the trauma surgery fellow presented closer results to the gold standard, showing the potential of the TV regarding image evaluation of non-radiologists.

Since the presented work focused on the potential benefits of a new prototype technique for the evaluation of multiple trauma CT, the primary aim was the recording of time parameters for reading the images and the number of correct diagnoses compared to the gold standard was considered as secondary aim. Of course in general the number of correct diagnoses stated using the new technique is of even more importance compared to simple time parameters, however these time parameters are relatively useful for determining the efficacy and potential benefit regarding a further step in the evaluation of this new prototype of course including correct diagnosis making.

### Design and functionality

In evaluating the design and functionality of the TV, it can be stated that the TV provides a good overview of the trauma CT scan. The overall scheme shown on the start display (Fig. 2) provides an orientation of all regions of interest. The possibility to choose among the different examined body parts (i.e. head/neck, abdomen etc.) is very well structured especially when non-radiologists want to take a first look on the multiple trauma CT before the radiologist provides his findings. This turns out to be useful since very often in multiple trauma patients, different medical disciplines are involved, so that each discipline gets the opportunity to have a fast look at his specific region of interest in all commonly used reformats of good image quality. Showing the different body parts on different monitors in the trauma room, with each specialist having the chance to focus on his region of interest, could not only accelerate the trauma assessment but also might improve the structure of the workflow with a possible benefit for the patient.

### Limitations of the study

There are several limitations of the study besides the retrospective character worth mentioning. In this study an early prototype was evaluated for a new concept for case presentation in trauma room care. Since the readers were not familiar using the TV, the evaluation using the

conventional secondary CT console was faster. Furthermore, the actual evaluation addresses only one part of the reading process and not the complete workflow starting with the image acquisition up to the determination of the final report. The TV presents with the advantage to automatically create reformats of individual body regions. However, the resulting benefit of shortening the time between CT scanning and image reading was not covered in this study. Additionally, the standard systems for primary and secondary reading of CT exams are either the PACS system or the PACS system in combination with the CT console. Hence, a setup comparing the TV and a PACS system for primary reading would complete the insights on the reading performance. Finally, only two readers analysed the CT-scans so that to obtain representative results a larger number of readers need to be acquired.

### Future trend

In order to overcome the limitations of the actual study, future studies are planned to investigate the effect of the complete reading workflow from image acquisition to the final diagnosis. In this context in terms of improving the reading workflow the Trauma Viewer will be integrated into the trauma room management, so that in future, all needed reformats will be automatically available in the trauma room, right when the patient receives his multiple trauma CT scan.

## Conclusions

In conclusion, reading trauma CT exams using the presented early prototype TV takes on average 1.15 min longer than reading at the well-known conventional secondary CT console, while there is no significant difference in the resulting diagnostic accuracy. Time delay using the TV can be adequately explained by the reader’s inexperience using this new modality and by the additional information in terms of multiple reformats, such as 3D reconstructions, provided by the TV in comparison to the conventional secondary CT console.

Automatically created reformats are assumed to have the potential to improve the diagnostic speed in the treatment of severely injured patients in the trauma room.

## Abbreviations

AIS: Abbreviated Injury Scale
CT: Computed Tomography
MPRs: Multiplanar reconstructions
MSCT: Multislice CT
TV: Trauma Viewer
